# Supplementary material for: Targeting the latent cytomegalovirus reservoir with an antiviral fusion toxin protein
Source: Nat Commun. 2017 Feb 2;8:14321. doi: 10.1038/ncomms14321 (PMC5296658; doi:10.1038/ncomms14321)
Supplement: Supplementary Information — Supplementary Figures [file ncomms14321-s1.pdf]

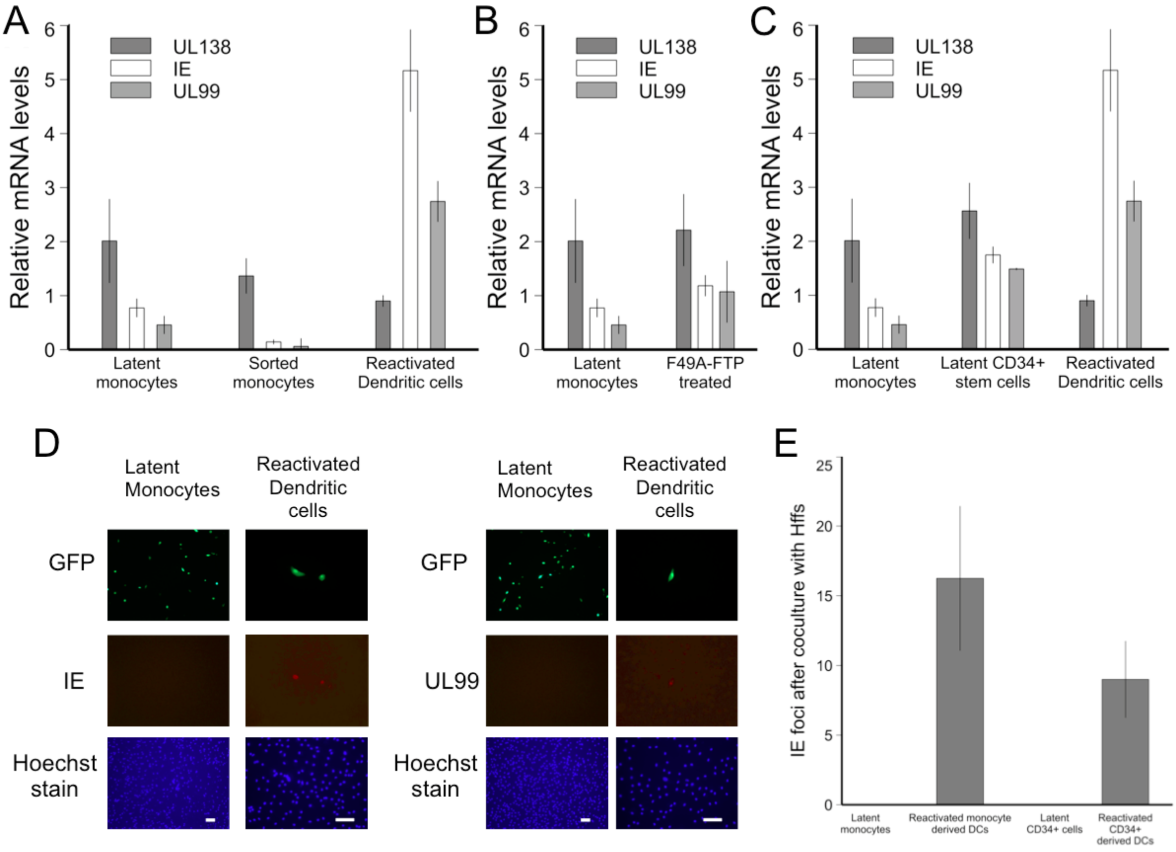

A
B
C
Relative mRNA levels
UL138
IE
UL99
Latent monocytes
Sorted monocytes
Reactivated Dendritic cells
F49A-FTP treated
Latent CD34+ stem cells
D
Latent Monocytes
Reactivated Dendritic cells
GFP
IE
UL99
Hoechst stain
E
IE foci after coculture with Hffs
Reactivated monocyte derived DCs
Latent CD34+ cells
Reactivated CD34+ derived DCs

Supplementary Fig. 1: Infected monocytes and CD34+ cells display hallmarks of latent infection, as do monocytes which were sorted by FACS. A) Monocytes were latently infected with SV40-GFP-TB40E at an MOI of 5, which led to a mean average of 12.2% latently infected cell, isolated by fluorescence activated cell sorting four days post infection, and RNA was harvested immediately after sorting. RT-qPCR analysis of this RNA demonstrated that sorted monocytes express UL138 (a latency associated gene) more so than the lytic genes: immediate early and UL99. Data was normalised to GAPDH RNA. This was compared to reactivated, monocyte-derived, mature dendritic cells, the RNA from these was harvested 4 days post terminal differentiation by LPS treatment, as a positive control for a lytic transcription profile. Means and error bars (showing standard deviations) were generated from three measurements. B) This same analysis was applied to monocytes, latently infected at an MOI of 5, and treated with F49A-FTP 24 hours after infection, for 72 hours. RNA was then harvested and analysed, demonstrating that F49A-FTP does not trigger virus reactivation. Means and error bars (showing standard deviations) were generated from three measurements. C) We also confirmed that our latently infected CD34+ cell population was, indeed, latently infected by harvesting RNA four days post infection and similarly comparing levels of latency-associated UL138 RNA to lytic IE and late viral gene expression in these cells. Means and error bars (showing standard deviations) were generated from three measurements. D) To demonstrate that low level detection of IE and UL99 by RT-qPCR in monocytes corresponded to limited protein expression, monocytes and reactivated dendritic cells were stained for IE and UL99 (pp28) and detected with a red fluorescent secondary antibody, either 4 days post infection or 4 days post terminal differentiation by LPS treatment. This demonstrated that UL99 and IE are not detectable in infected monocytes, but are expressed after differentiation and reactivation of these latently infected cells. White bars indicate 50μM scale. E) Monocytes or CD34+ stem cells were infected with HCMV and either differentiated with cytokine treatment and matured, or mock treated. After this, cells were co-cultured with HFFs, and incubated for 2 weeks. Cultures were then stained for IE gene expression, and IE positive foci counted. Data show mean and standard deviations from three independent experiments.

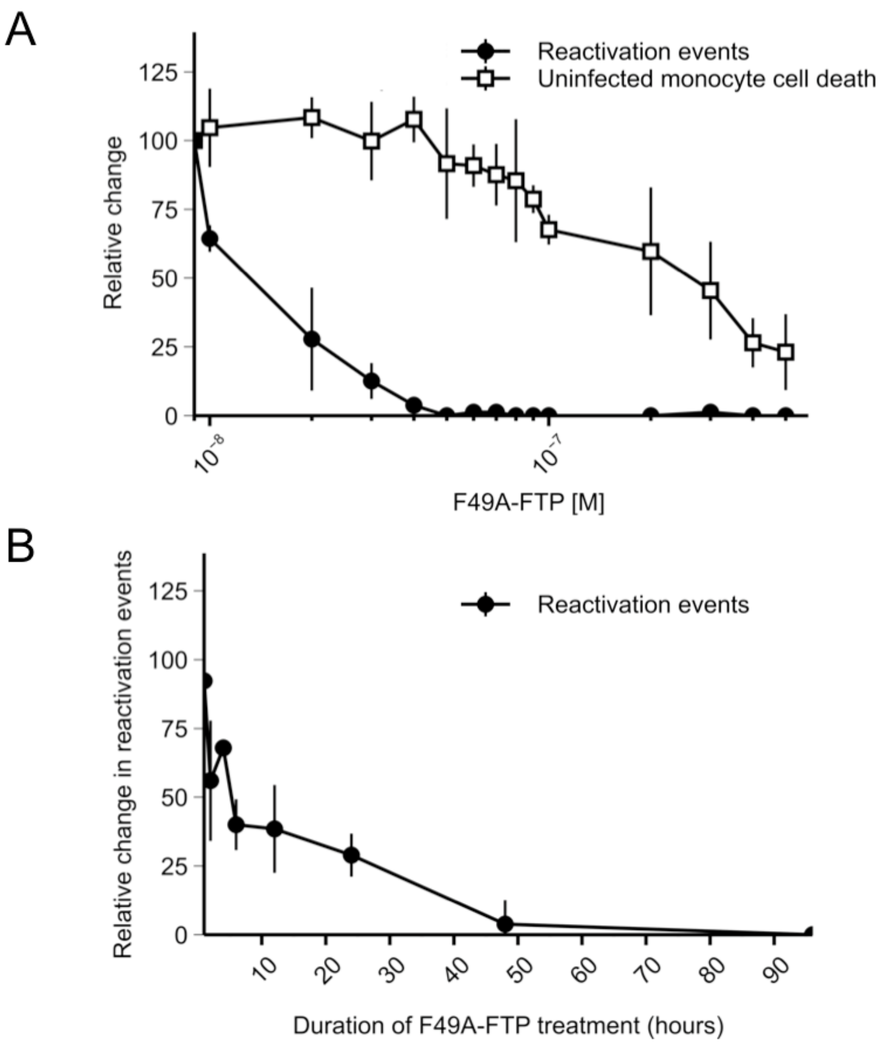

Supplementary Fig. 2: F49A-FTP kills latently infected monocytes specifically compared to uninfected monocytes. A) Titrations of F49A-FTP on monocytes that were infected at an MOI of 5, which led to a mean average of 5.7% of cells latently infected. Titration of F49A-FTP showed that reactivation events were greatly reduced before significant reductions in monocyte viability. A concentration of $5x10^{-8}$ M was chosen for subsequent experiments to optimise F49A-FTP efficacy while minimising monocyte cell death. Means and error bars (showing standard deviations) were generated from four independent experiments. B) monocytes, which were latently infected at an MOI of 5 with SV40-GFP-TB40E, which led to a mean average of 9.0% of cells latently infected, and were incubated with F49A-FTP for increasing amounts of time, followed by differentiation and quantification of subsequent reactivation events, in order to establish a minimum incubation time for the toxin. This established that a 48 hour incubation was sufficient to clear latently infected cultures of HCMV. Means and error bars (showing standard deviations) were generated from three independent experiments.

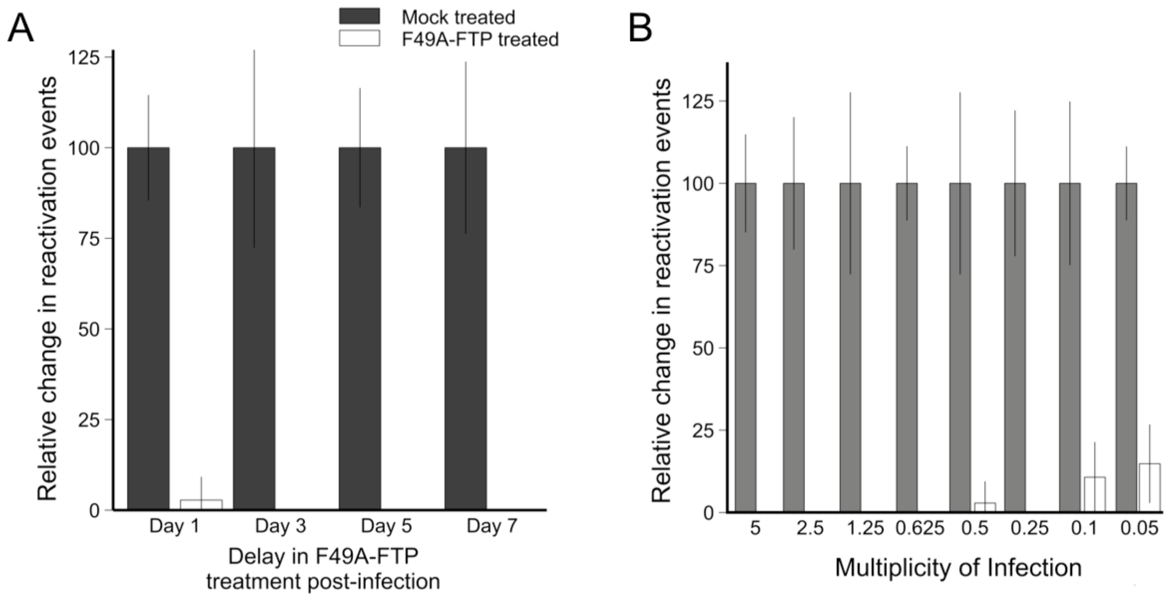

Supplementary Fig. 3: Delaying treatment of latently infected monocytes with F49A-FTP does not alter its efficacy at clearing latent HCMV; F49A-FTP is also effective at clearing virus at low multiplicities of infection. A) Monocytes were isolated and infected with SV40-GFP-TB40E wild type virus at a predicted MOI of 5, which led to a mean average of 7.6% of cells latently infected. Cultures were then treated with F49A-FTP between one and seven days post infection. After this, latently infected cells were reactivated by differentiation and maturation to mDCs. These monocyte-derived, mature dendritic cells were then co-cultured with fibroblasts for two weeks, and the number of reactivated IE foci were detected by immunoflourescent staining. In every case, F49A-FTP was able to clear latent virus effectively. B) Monocytes were infected with increasing dilutions of a stock of SV40-GFP-TB40E wild type virus (as shown on the x axis, based on infection of RPE-1 cells), which corresponded to estimated percentages of latently infected cells: 9.1%, 4.8%, 3.2%, 1.3%, 0.76%, 0.47%, 0.15% and 0.08% respectively. These were treated with F49A-FTP 24 hours post infection. After this, latently infected cells were reactivated by differentiation and maturation to mDCs. These monocyte-derived, mature dendritic cells were then co-cultured with fibroblasts for two weeks, and the number of reactivated IE foci were detected by immunoflourescent staining. For lower MOIs, an increased number of monocytes were infected in order to keep reactivation events similar across all experiments. The total reactivation events measured, for the untreated control experiments, from left to right, were: 52, 48, 53, 43, 45, 47, 36, and 35 respectively. For F49A-FTP treated cultures infected with MOIs of 0.5, 0.1 and 0.05, the number of reactivation events was 1, 4 and 5, respectively. F49A-FTP was therefore still very effective at clearing latent virus, although a slight drop in efficacy was noticable at MOIs of 0.1 and 0.05. Means and error bars (showing standard deviations) were generated from three independent experiments.

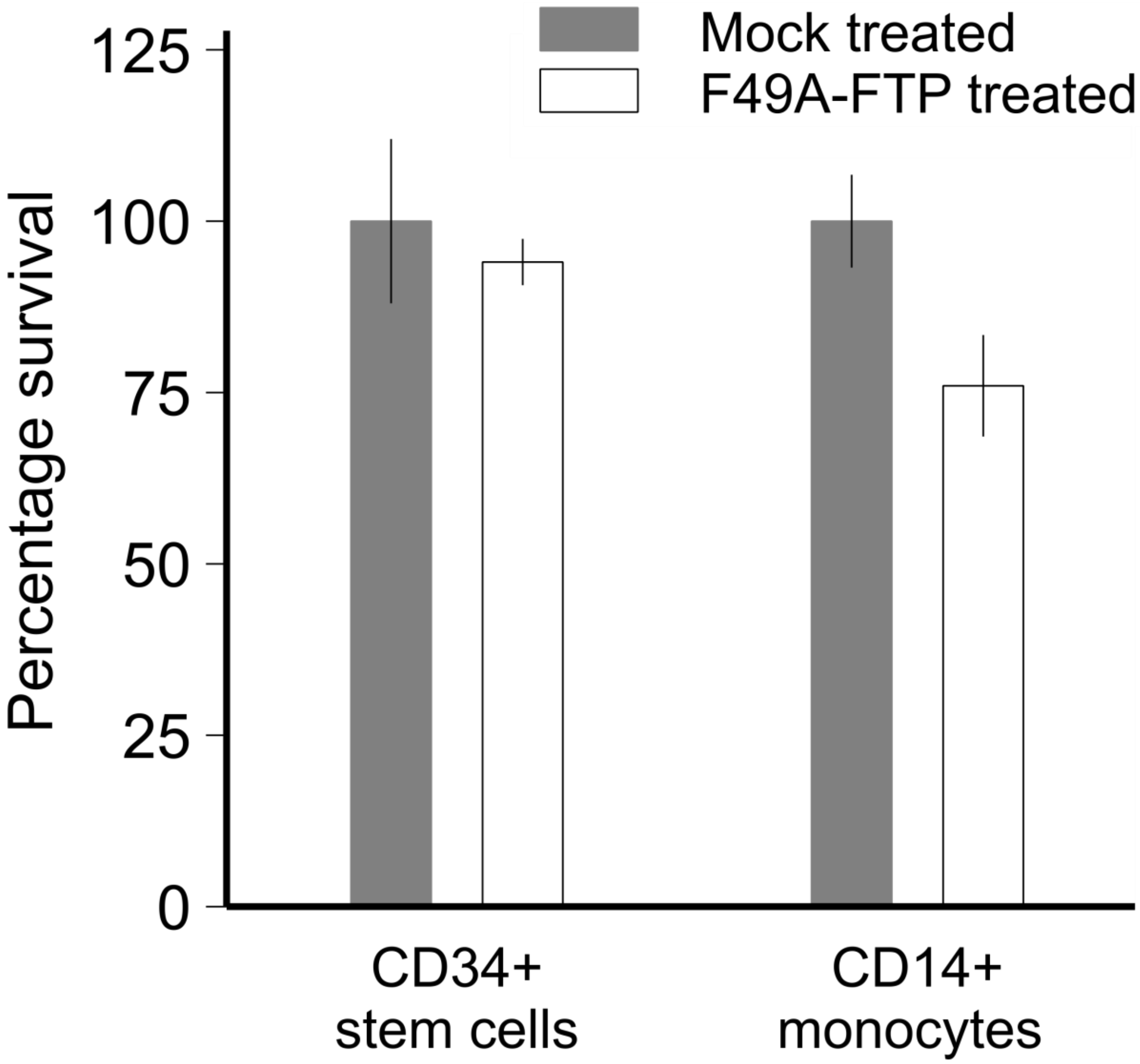

Supplementary Fig. 4: F49A-FTP has residual toxicity towards uninfected monocytes, and no detectable toxicity towards CD34+ progenitor cells. Uninfected monocyte and CD34+ progenitor cells were treated with F49A-FTP. After 72 hours, cells were stained with trypan blue and dead (blue) cells were counted and calculated as a percentage of total cells. For this experiment, $1x10^{-7}$M F49A-FTP was used, to demonstrate differences in sensitivity between CD34+ progenitor cells and CD14+ monocytes. Means and error bars (showing standard deviations) were generated from four independent experiments.

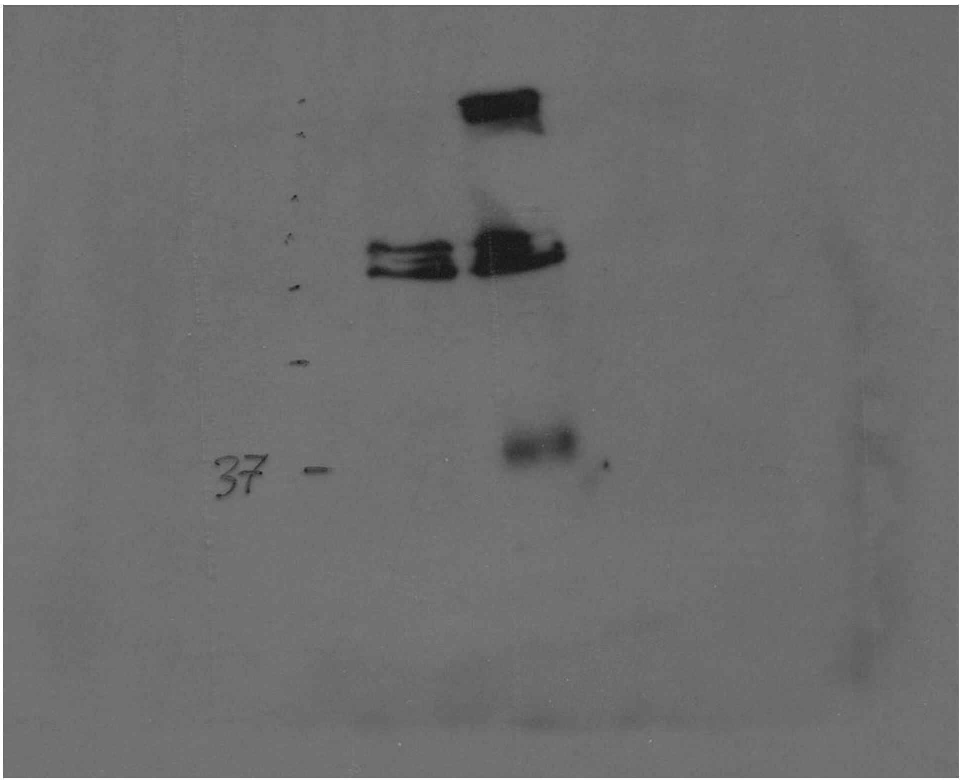

Supplementary Fig. 5: THP-1 cells transduced with an N terminally tagged US28 construct, in a lentiviral vector, express US28 protein, which can be detected by immunoblot again. THP-1 cells expressing HA-US28 were generated by lentiviral transduction and puromycin selection. A) western blots of cell lysates from transduced THP-1 cells against the N-terminal HA tag on HA-US28 (right lane), control cells were mock transduced (left lane). Molecular markers indicate molecular weights for 37 kDa (indicated), 50 kDa, 75 kDa, 100 kDa, 150kDa and 200kDa, from the bottom to the top of the gel. HA-US28 protein, at 37 kDa, can only be detected in transduced cells A non-specfic band at 75kDa can be used as a loading control. At 150-200 kDa, a band can be seen which we believe to be precipitated US28 protein, which did not enter the polyacrylamide gel.
